# Associations between Food Policy Councils and Policies That Support Healthy Food Access: A National Survey of Community Policy Supports

**DOI:** 10.3390/nu13020683

**Published:** 2021-02-20

**Authors:** Samantha J. Lange, Larissa Calancie, Stephen J. Onufrak, Katherine T. Reddy, Anne Palmer, Amy Lowry Warnock

**Affiliations:** 1 Oak Ridge Institute for Science and Education (ORISE) Research Participation Program, Oak Ridge, TN 37870, USA; 2 Centers for Disease Control and Prevention, Division of Nutrition, Physical Activity, and Obesity, Atlanta, GA 30341, USA; seo5@cdc.gov (S.J.O.); hwf9@cdc.gov (A.L.W.); 3 ChildObesity180, Friedman School of Nutrition Science and Policy, Tufts University, Boston, MA 02111, USA; larissa.calancie@tufts.edu; 4 McKing Consulting Corporation, Atlanta, GA 30341, USA; iet3@cdc.gov; 5 Johns Hopkins Center for a Livable Future, Johns Hopkins Bloomberg School of Public Health, Baltimore, MD 21202, USA; apalmer6@jhu.edu

**Keywords:** food policy council, local government, healthy food access, policy, municipality

## Abstract

Food policy councils (FPCs) are one form of community coalition that aims to address challenges to local food systems and enhance availability, accessibility, and affordability of healthy foods for local residents. We used data from the 2014 National Survey of Community-Based Policy and Environmental Supports for Healthy Eating and Active Living, a nationally representative survey of US municipalities (*n* = 2029), to examine the prevalence of FPCs and cross-sectional associations between FPCs and four types of supports for healthy food access (approaches to help food stores, practices to support farmers markets, transportation-related supports, and community planning documents). Overall, 7.7% of municipalities reported having a local or regional FPC. FPCs were more commonly reported among larger municipalities with ≥50,000 people (29.2%, 95% Confidence Interval (CI): 21.6, 36.8) and western region municipalities (13.2%, 95% CI: 9.6, 16.8). After multivariable adjustment, municipalities with FPCs had significantly higher odds of having all four types of supports, compared to those without FPCs (adjusted odds ratio (aOR) range: 2.4–3.4). Among municipalities with FPCs (*n* = 156), 41% reported having a local government employee or elected official as a member, and 46% had a designated health or public health representative. Although FPCs were uncommon, municipalities that reported having a local or regional FPC were more likely to report having supports for healthy food access for their residents.

## 1. Introduction

Policies, programs, and organizational practices that support healthy food access can improve diet quality and may help prevent obesity and promote self-management of diet-related chronic diseases on a population level [1]. These policies can enhance community food environments by promoting “the healthy choice as the easy choice” for individuals and families [2]. Such policies and practices not only have the potential to reduce risk for chronic diseases, but may also support economic development, biodiversity, equitable food systems, and promote community resilience [2,3]. Local governments have the legal authority to consider policies and practices instituted by governmental organizations, such as planning departments, as options for promoting health within their jurisdiction. For example, they may adopt a healthy food procurement policy, which can enhance availability of healthy food and beverage options in public parks [4]; or implement broader food service guidelines, which may require vendors in government facilities, hospitals, and other worksites to meet minimum nutrition standards [5]. Other types of healthy food policies may be used to incentivize the establishment of supermarkets; enhance accessibility to farmers markets; increase availability of healthy, affordable foods in small convenience or corner stores; improve transportation options to healthy food retailers; or support local agriculture and land protections [6]. Specific municipal-level policy examples may be found via the Healthy Food Policy Project’s online, searchable database (https://healthyfoodpolicyproject.org/) (accessed on 12 February 2020).

Food policy councils (FPCs) are one form of community coalition that can facilitate the adoption and implementation of policies that support healthy food access. FPCs are an “organized group of stake-holders that may be sanctioned by a government body, or exist independent of government, which work to address food systems issues and needs at the local (city/municipality or county), state/pro-vincial, regional, or tribal levels” [7]. FPCs exist across the United States of America, Canada, Tribal and First Nations, as well as in Australia and Europe [8]. FPC members may include a variety of stakeholders representing different sectors within the food system such as regional farmers, large-scale purchasers, representatives from food banks or other charitable feeding programs, public health practitioners, local and state government representatives, and interested citizens. FPCs bring together these diverse stakeholders to plan for collective action to improve communities [9,10]. FPCs have long been considered an important strategy to promote healthy eating by the Centers for Disease Control and Prevention (CDC) [11].

Some research has been conducted on FPCs, but there are gaps in the existing literature. Since 2009, the CDC has tracked the number of active local- and state-level FPCs as part of State Indicator Reports on Fruits and Vegetables as a potential approach to increase access to nutritious foods [12]. In 2015, the International City/County Management Association and Michigan State University’s Center for Regional Food Systems conducted a survey of more than 2000 local governments to understand how they influenced food systems in their areas [13]. Approximately 13% of respondents indicated that citizen commissions or advisory boards were the primary driver of efforts to improve the food system in their communities. Furthermore, 19% of respondents reported representation from local government staff on such FPCs. Some local governments actively support local food systems and accessibility to healthy foods via food system plans, policies, and programs (e.g., increasing the purchasing power of food assistance benefits through bonus vouchers or providing land for community gardens). Although anecdotal evidence and case studies have documented the work of specific FPCs, we are unaware of any research that measures the association between the presence of FPCs and specific municipal-level policies or practices that support healthy eating using a nationally representative sample.

The aims of this study were: (1) to use reported information from municipalities to document the national prevalence of local or regional FPCs overall and by municipal characteristics, and (2) to examine the associations between the presence of FPCs and four types of policies and practices that support healthy food access in food stores, healthy food access in farmers markets, transportation to healthy food retailers, and community planning related to farmers markets, community gardens, and agricultural land. We used existing data from the 2014 US Survey of Community-Based Policy and Environmental Supports for Healthy Eating and Active Living (CBS HEAL).

## 2. Methods

The CBS HEAL survey was conducted by the CDC from May through September 2014. It sought to identify the presence of local policies and practices among US municipalities to support healthy living for community residents. The sampling frame was based on the US Census Bureau’s 2007 Census of Governments, which lists municipal governments (hereafter referred to as “municipalities”) by state [14]. Municipalities of fewer than 1000 people were excluded from the sample pool based on a pilot study that showed that smaller communities were less likely to have policies and practices to support healthy eating and active living [15]. To create a nationally representative sample of municipalities, sampling was stratified by geographic census region (West, Northeast, South, and Midwest) and by percentage of area urbanized (using the 30th percentile as a cut-off point), then sorted by population size with a fixed sampling interval. Among 10,205 eligible municipalities, the final sample included 4484 municipalities. The survey response rate was 45% (*n* = 2029).

The CBS HEAL survey gathered information on the existence of certain policies and practices implemented by municipalities to promote healthy eating and active living. The survey was developed based on literature reviews, scans of existing national policy databases, and input from state health department grantees and other topic experts. Additional information regarding survey development has been described elsewhere [15]. The survey was sent to city or town planners, managers, or persons with similar responsibilities. Respondents completed the survey with assistance from other municipal officials, if needed, via a secure website or a paper-based version.

To assess the presence of FPCs, respondents were asked, “Does your jurisdiction have a local or regional food policy council, food security coalition, or similar entity? A food policy council is a council that brings together stakeholders from diverse food-related sectors in a specific geographical area to examine how the food system is operating in that area and to develop recommendations for improvement.” (Table 1). Having an FPC was defined by a response of “Yes” to this question; responses of “No” and “Don’t know” were considered as not having an FPC. If the respondent answered “Yes”, follow-up questions included, “Is a local government employee or elected official a member of the food policy council, food security coalition, or similar entity?” and “Is there a designated health/public health representative on the food policy council, food security coalition, or similar entity?”; response options were “Yes”, “No”, or “Don’t know”.

Respondents were also asked whether their communities had various policies or practices to support increased access to healthy foods (Table 1). We grouped these questions into four categories based upon topic modules inherent in the survey instrument: (1) approaches to open new supermarkets or help existing convenience or corner stores sell healthy foods (food stores category); (2) practices to support farmers markets, farm stands, and green/produce carts (farmers markets category); (3) transportation-related supports for accessing supermarkets, other full-service grocery stores, or farmers markets (transportation-related category); and (4) consideration of farmers markets/community gardens and agricultural land in community planning documents (planning category). For each of the four categories, the outcome variable of having any policy or practice to support healthy food access was defined by a response of “Yes” to at least one question in the respective category. The referent group comprised municipalities that either responded “No”, “Don’t know”, or were missing a response to all questions in the respective category.

Control variables used in this analysis included population size, urban/rural status, geographic region, median education level, poverty prevalence, and racial/ethnic composition. Municipal population size was obtained from the 2007 Census of Governments [14] and categorized into three levels: 1000–2499, 2500–49,999, and ≥50,000 people. Municipalities were considered urban if more than 50% of the population resided in areas defined as urban based on the 2010 US Census Urban Area to Place Relationship File [16]. Municipalities were also classified into the four geographic census regions: West, Northeast, South, and Midwest [17]. The 2009–2013 American Community Survey was used to define median education level of the population aged 25 years or older (≤high school diploma vs. ≥some college), percentage of the population living below the federal poverty line (FPL) (<20 vs. ≥20% to reflect persistent poverty as defined by the U.S. Department of Agriculture), and racial/ethnic composition (≤50 vs. >50% non-Hispanic white) for each municipality [18].

Ten (0.5%) of the 2029 municipalities who completed the survey were excluded because they were missing responses to the survey question about FPCs. The final analytic sample size was 2019 municipalities. The prevalence of having FPCs and associated 95% confidence intervals (CIs) were calculated overall and by population size, urban/rural status, geographic region, median education level, poverty prevalence, and racial/ethnic composition. Among municipalities with FPCs, we also calculated the proportion that reported having representation from (a) local government or (b) health/public health.

Chi-square tests assessed differences in the prevalence of having FPCs by municipality characteristics. A *p*-value of <0.05 defined statistical significance. Logistic regression models were run to obtain odds ratios of having an FPC adjusted for municipality characteristics. We also used logistic regression to examine associations between having an FPC and the four categories of supports for increasing access to healthy foods (food stores, farmers markets, transportation-related, and planning) and their sub-questions, adjusted for municipality characteristics. Because 11% of municipalities responded “Don’t know” to the FPC question, a sensitivity analysis was run to test whether results differed when the referent group included responses of “No” only vs. “No” or “Don’t know” (i.e., the primary analysis). Analyses were weighted to account for the complex survey design, including unequal probabilities of selection and varying non-response rates, using region and urban/rural status strata to define weighting classes. All analyses were conducted using survey procedures in SAS version 9.4 (SAS Institute Inc., Cary, NC, USA).

## 3. Results

The majority of municipalities in this sample had a population of 2500–49,999 people (58.4%), were urban (74.8%), had a median education level of some college or higher (55.6%), had <20% poverty prevalence (69.8%), and had a racial/ethnic distribution of >50% non-Hispanic white (86.6%).

Among municipalities with at least 1000 residents in the USA in 2014, 7.7% reported having a local or regional FPC, food security coalition, or similar entity (Table 2). Prevalence of FPCs varied significantly by some municipal characteristics. Having an FPC was more common among municipalities with 50,000 or more people (29.2%) compared to those with 2500–49,999 (7.0%) or fewer than 2500 people (4.3%) (Table 2). After multivariable adjustment, municipalities with ≥50,000 people had 7.7 (95% CI: 3.4, 17.7) times higher odds of having an FPC compared to municipalities with <2500 people. FPCs were also more common among urban municipalities compared to rural (8.7% vs. 4.2%) and municipalities with <50% non-Hispanic white populations compared to those that were majority non-Hispanic white (10.8% vs. 7.1%); however, these differences did not remain significant after adjustment for other municipality characteristics. Municipalities in western states had approximately two times higher adjusted odds of having FPCs than municipalities in southern states (adjusted odds ratio (aOR): 1.9, 95% CI: 1.2, 3.2). Sensitivity analysis comparing “Yes” vs. “No” yielded aORs that were similar in magnitude and statistical significance to the aORs comparing “Yes” vs. “No” or “Don’t know” (data not shown).

Having an FPC was significantly associated with municipal-level policies or practices to improve access to healthy foods. Among municipalities with FPCs (*n* = 156), nearly all (96.9%) reported having at least one policy support for healthy food access, compared to 84.9% of municipalities without FPCs (Table 3). After multivariable adjustment, municipalities with FPCs had significantly higher odds of having any supports (aOR: 4.1, 95% CI: 1.6, 10.4), supports for food stores (aOR: 3.3, 95% CI: 2.3, 4.7), supports for farmers markets (aOR: 3.3, 95% CI: 2.0, 5.5), transportation-related supports to increase access to healthy foods (aOR: 2.4, 95% CI: 1.6, 3.5), and objectives in community planning documents (aOR: 3.4, 95% CI: 2.1, 5.5), compared to municipalities without FPCs. Table 3 contains aORs and corresponding 95% CIs for the specific supports in each of the above categories. Except for vans or shuttles as transportation to healthy food retailers, all of the specific policies and practices examined within each category were also significantly associated with the presence of FPCs.

Among the 156 municipalities that reported having an FPC, 41% reported that they had a local government employee or elected official as a member of the FPC, and 46% reported that they had a designated health or public health representative on the FPC (data not shown). Nearly one third of municipalities with an FPC (31.7%, *n* = 50) reported having representation from both the local government and health/public health sectors on their FPCs.

## 4. Discussion

Using data from a nationally representative survey of 2029 municipalities, this study estimated the national prevalence of FPCs by municipal and regional characteristics and examined the association of FPCs with local supports for healthy food access in communities across the USA. We found significant associations between the presence of FPCs and local policies and practices that support healthy food access, independent of municipal population size, urban/rural status, geographic region, median education level, poverty prevalence, and race/ethnicity. These supports can enhance community food environments by improving availability of and accessibility to healthy foods, which may also promote healthy eating and prevention of diet-related chronic diseases [1]. FPCs and similar community coalitions serve as unique avenues to initiate, support, or enhance local agendas for improving food systems and environments. They offer social infrastructure that connects key stakeholders, organizations, and decision-makers, enabling information, priorities, and trust to flow among these stakeholders to advance community priorities [2,19]. This social infrastructure may help explain the observed associations between FPCs and local supports for healthy food access. Our findings suggest that municipal governments may consider initiating, supporting, or collaborating with FPCs to increase the adoption and implementation of policies and practices that support healthy food access for community residents.

The policies and practices examined in this study align with the top priorities reported by FPCs in a recent publication by the Food Policy Network (FPN). According to FPN’s most recent report, the top-ranked priorities for FPCs in the USA were increasing access to healthy foods, economic development, and hunger relief [7]. Priorities related to increasing healthy food access included healthier vending, Supplemental Nutrition Assistance Program (SNAP) incentives at farmers markets, and healthy food retail financing [7]. Additionally, a study that examined FPC policy, systems, and environmental initiatives found that 54% of 317 initiatives were related to healthy food access [3]. Common initiatives included supportive zoning for farm stands and facilitating the use of Electronic Benefits Transfer (EBT) at farmers markets, which we assessed in the present study [20]. Promoting equity is another priority for an increasing number of FPCs, including a coalition in Australia that works to increase access to healthy foods in a rural area [21], and the Prince George County Council in Maryland, USA, which pursued policies to curtail unhealthy food access in minority communities [22]. Future studies may investigate whether FPCs can facilitate municipal-level policies and practices that support equitable healthy food access.

Our findings suggest differences in the prevalence of FPCs by geography and other municipal characteristics. We found a significantly higher proportion of municipalities reporting FPCs in western states (13.2%), compared to southern states (5.9%). This finding is corroborated by the FPN’s report, which showed similar regional differences among reporting FPCs [9]. Furthermore, municipalities with ≥50,000 people were significantly more likely to report having an FPC than smaller ones. This is consistent with other studies, which found that large municipalities are more likely than small ones to work on strategies to enhance healthy food access [23,24]. These differences may reflect more financial resources, personnel, or political capital to implement healthy food policies in larger municipalities vs. smaller ones, or other factors.

The overall prevalence of municipalities reporting a local or regional FPC is low (7.7%). According to FPN’s 2018 report, the number of FPCs continues to grow each year, but at a relatively slow rate. For example, at the end of 2017, there were 341 FPCs in the USA and Canada, up from 329 in 2016. Further, the majority of FPCs in the USA (71%) reported operating at the “local” level, including 36% at the county level, 20% at the city/municipal level, and 15% at both. An additional 20% are regional (multi-county or multi-state), and 8% operate at the state level [7]. US FPCs, especially newer ones, are prone to county level jurisdiction. Heterogeneity exists in the structure and type of jurisdiction of FPCs; differences in how FPCs are defined may have influenced interpretation of our survey question on FPCs. Because we specifically asked about local or regional FPCs, it is plausible that some municipalities did not report having an FPC if their municipality belonged to a broader, county-level FPC. As such, we may have missed some of the influence of county-level FPCs on policies and practices that support healthy food access in smaller municipalities. Additionally, municipalities may face bureaucratic challenges or other barriers that influence the development and operation of FPCs.

In our study, nearly 50% of municipalities with FPCs reported having a health or public health representative on the council. This proportion is lower than what was reported in the FPN’s annual survey of councils; approximately 85% reported having a public health department representative and 65% a healthcare representative [7]. Nonetheless, our results illustrate that it is common for public health professionals to sit on local FPCs. FPCs and public health departments often have synergistic goals related to increasing access to healthy foods, which can be mutually beneficial to the missions of each organization. Local FPCs are well-positioned to leverage public health data and food system data to create a targeted plan of action. As synergistic opportunities arise, FPCs and public health departments can build upon collaborative efforts to improve the local economy through agriculture, distribution, and retail, while enhancing access to healthier foods for citizens.

The findings from this study are subject to limitations. First, we relied on self-reported information from city managers, planners, and similar representatives; this may have resulted in misclassification as we were unable to verify the existence of reported FPCs or policies and practices. For example, respondents may have under-reported FPC prevalence if they were not personally familiar with their community’s FPC. Second, these findings have somewhat limited generalizability as they only apply to municipalities with ≥1000 people. Third, the survey had a modest response rate; however, we incorporated weighting procedures into our statistical analyses to account for the probability of non-response. Fourth, this study is cross-sectional and cannot assess a causal link between FPCs and the existence of healthy food supports. Fifth, the data from the survey were collected six years ago and the prevalence of FPCs and various policy supports may have since changed; however, because this is the only survey in existence that examines supports for healthy eating and active living among a nationally representative sample of US municipalities, the results are still a valuable addition to the literature. Finally, although we assessed associations between FPCs and municipal supports in four important categories (food stores, farmers markets, transportation, and planning), FPCs also work on additional issues that were not measured in this survey, such as food procurement, farm to school/early care and education programs, and food waste and recovery policies. Future research efforts may investigate the temporality of associations between FPCs and policies that support healthy food access, acknowledging that FPC efforts may change over time due to a variety of factors such as leadership or funding. Further identification and understanding of the factors that influence FPC efforts may also be an avenue for future work.

## 5. Conclusions

Although FPCs were not commonly reported by US municipalities, municipalities that reported having a local or regional FPC were more likely to report having local policies and practices to support healthy food access for their residents. To our knowledge, this is the first study to measure the association between local or regional FPCs and specific local supports for grocery and convenience stores, farmers markets, transportation to healthy food retailers, and community planning using a nationally representative sample of US municipalities. FPCs may help organize key stakeholders, identify high-priority needs, and advance community-driven action that supports healthy food access and a broader range of effects, such as equitable food systems and economic development, and community resilience. Our findings support the notion that communities wishing to adopt and implement policies and practices that support healthy food access may benefit from establishing, supporting, or collaborating with an FPC.

## Figures and Tables

**Table 1:** Survey questions about food policy councils (FPCs) and municipal policies and practices that support access to healthy foods, National Survey of Community-Based Policy and Environmental Supports for Healthy Eating and Active Living, United States, 2014.

Food Policy Council
Does your jurisdiction have a local or regional food policy council, food security coalition, or similar entity? a) Is a local government employee or elected official a member of the food policy council? b) Is there a designated health/public health representative on the food policy council, food security coalition, or similar entity?
New Supermarkets or Existing Convenience or Corner Stores
Supermarket Supports: Does your local government currently use any of the following approaches to encourage supermarkets and other full-service grocery stores to open stores? a) Tax incentives (tax abatement, tax credit, or property tax exemption)? b) Grant or loan programs? c) Zoning or ordinance requirement fee waivers or streamlined processes for obtaining health and food safety permits and licenses? d) Programs to link store openings to broader neighborhood revitalization projects (improvements to lighting, signage, safety, or walkability in the surrounding commercial corridor)? e) Other incentive programs? Convenience/Corner Store Supports: Does your local government provide any of the following to help convenience or corner stores sell healthier foods? a) Grant or low-interest loan programs to purchase/upgrade store equipment or furnishings to properly store and sell healthful foods and beverages (for example, fresh produce, low-fat milk, or whole grains)? b) Technical assistance or training programs that increase a store’s ability to sell healthier foods (for example, assistance with marketing, promotion materials, and/or product placement)? c) Programs to link stores to broader neighborhood revitalization projects (for example, improvements to lighting, signage, safety, or walkability in the surrounding commercial corridor)? d) Other types of incentive programs that help convenience or corner stores sell healthier foods?
Farmers Markets, Farm Stands, & Green/Produce Carts
Permitting/Financial Supports: Does your local government have any policies related to farmers markets, farm stands, or green/produce carts that. a) Allow the sale of fresh produce on city property? b) Streamline processes for obtaining health or food safety permits and licenses? c) Extend waivers of required business permits or retail licensing fees or taxes? d) Provide funds or in-kind services for personnel, signage, or advertising? e) Encourage opening in areas lacking supermarkets or full-service grocery stores? Support for EBT: Does your local government provide funding for Electronic Benefits Transfer (EBT) machines or provide technical assistance on how to obtain or use EBT machines at local farmers markets, farm stands, or green/produce carts?
Transportation-Related Supports
Dedicated Vans/Shuttles: Does your local government have a policy that supports dedicated transportation (e.g., community vans or shuttle buses) to supermarkets, other full-service grocery stores, or farmers markets for these residents? Consideration of Accessibility: Does your local government consider accessibility to supermarkets or other full-service grocery stores in their assessment of public transportation routes?
Community Planning Documents
Does your local government have any of the following objectives included in the (comprehensive/general/master) plan(s)? a) Supporting farmers markets or community gardens? b) Preserving land for agricultural uses?

**Table 2:** Prevalence, 95% confidence intervals, and adjusted odds of having a local or regional food policy council (FPC), food security coalition, or similar entity among US municipalities, National Survey of Community-Based Policy and Environmental Supports for Healthy Eating and Active Living, United States, 2014.

Municipality Characteristic	*n*	Yes, % (95% CI)	*p*-Value ^b^	aOR ^c^ (95% CI)
All Municipalities ^a^	2019	7.7 (6.5, 8.8)	--	--
**Population Size**				
<2500 people	717	4.3 (2.8, 5.8)	**<0.0001**	Ref
2500–49,999 people	1160	7.0 (5.6, 8.5)		1.5 (0.7, 3.1)
≥50,000 people	142	29.2 (21.6, 36.8)		**7.7 (3.4, 17.7)**
**Urban/Rural Status**				
Rural (≤50% urban)	538	4.2 (2.5, 5.9)	**0.0006**	Ref
Urban (>50% urban)	1481	8.7 (7.3, 10.2)		1.3 (0.6, 2.8)
**Geographic Region**				
South	706	5.9 (4.1, 7.6)	**0.0012**	Ref
Midwest	745	7.6 (5.7, 9.5)		1.6 (1.0, 2.6)
Northeast	233	6.5 (3.3, 9.7)		1.2 (0.7, 2.3)
West	335	13.2 (9.6, 16.8)		**1.9 (1.2, 3.2)**
**Median Education Level**				
≤High school	890	7.0 (5.3, 8.7)	0.3749	Ref
≥Some college	1129	8.1 (6.5, 9.6)		0.8 (0.6, 1.2)
**Poverty Prevalence**				
<20% below FPL	1409	7.1 (5.8, 8.4)	0.2029	Ref
≥20% below FPL	610	8.7 (6.5, 11.0)		1.3 (0.9, 1.9)
**Race/Ethnicity**				
>50% non-Hispanic white	1751	7.1 (5.9, 8.3)	**0.0354**	Ref
≤50 non-Hispanic white	268	10.8 (7.1, 14.5)		1.0 (0.6, 1.7)

FPC = food policy council, FPL = federal poverty line, CI = confidence interval, aOR = adjusted odds ratio, ^a^ Excludes *n* = 10 municipalities with missing responses to the survey question "Does your jurisdiction have a local or regional food policy council, food security coalition, or similar entity?" ^b^ Calculated using a Chi-square test for differences in reported prevalence of FPC, ^c^ Logistic regression adjusted for municipal population size, urban/rural status, geographic region, median education level, poverty prevalence, and race/ethnicity. Referent group was those municipalities who reported "No" or "Don’t know" to the food policy council question. The bold indicate statistical significance.

**Table 3:** Prevalence, 95% confidence intervals, and adjusted odds ratios of reported policies or practices to improve access to healthy foods in municipalities with and without local or regional food policy councils (FPCs), National Survey of Community-Based Policy and Environmental Supports for Healthy Eating and Active Living, United States, 2014.

	Overall Prevalence ^a^		Prevalence among Those with FPCs (*n* = 156)		Prevalence among Those without FPCs (*n* = 1631)		Adjusted Odds Ratio (FPC vs. no FPC ^b^)	
Type of Supports for Healthy Food Access	*n*	%	%	95% CI	%	95% CI	aOR	95% CI
Any	1538	85.9	**96.9**	**(94.1, 99.6)**	**84.9**	**(83.1, 86.6)**	**4.1**	**(1.6, 10.4)**
New Supermarkets or Existing Convenience or Corner Stores	665	36.9	**63.8**	**(56.3, 71.3)**	**34.4**	**(32.1, 36.7)**	**3.3**	**(2.3, 4.7)**
Supermarkets Supports	611	34.0	**54.3**	**(46.5, 62.2)**	**32.1**	**(29.8, 34.3)**	**2.6**	**(1.8, 3.6)**
Convenience/Corner Store Supports	242	13.6	**41.7**	**(33.9, 49.6)**	**11.0**	**(9.4, 12.5)**	**4.8**	**(3.3, 7.1)**
Farmers Markets, Farm Stands, & Green/Produce Carts	1209	67.6	**88.1**	**(83.0, 93.2)**	**65.9**	**(63.3, 68.0)**	**3.3**	**(2.0, 5.5)**
Permitting or Financial Supports for Farmers Markets	1201	67.1	**86.9**	**(81.6, 92.2)**	**65.3**	**(63.0, 67.6)**	**3.1**	**(1.9, 5.0)**
Support for EBT at Farmers Markets	104	5.8	**19.3**	**(13.1, 25.6)**	**4.5**	**(3.5, 5.6)**	**3.2**	**(1.9, 5.6)**
Transportation-Related Supports	518	29.0	**54.3**	**(46.4, 62.2)**	**26.6**	**(24.5, 28.7)**	**2.4**	**(1.6, 3.5)**
Dedicated Vans/Shuttles to Healthy Food Retailers	259	14.6	17.5	(11.6, 23.5)	14.4	(12.7, 16.1)	1.0	(0.6, 1.7)
Consideration of Accessibility in Public Transit Routes	409	22.7	**49.8**	**(41.9, 57.7)**	**20.1**	**(18.2, 22.1)**	**2.8**	**(1.9, 4.1)**
Community Planning Documents	1143	63.8	**86.9**	**(81.6, 92.2)**	**61.6**	**(59.2, 64.0)**	**3.4**	**(2.1, 5.5)**
Farmers Markets/Community Gardens	1013	56.6	**80.1**	**(73.9, 86.3)**	**54.4**	**(52.0, 56.8)**	**2.8**	**(1.8, 4.3)**
Preserving Land for Agricultural Uses	571	31.4	**48.2**	**(40.3, 56.1)**	**29.8**	**(27.6, 32.0)**	**1.9**	**(1.4, 2.8)**

FPC = food policy council, CI = confidence interval, aOR = adjusted odds ratio, EBT = electronic benefits transfer, odds ratios adjusted for municipality characteristics including population size, urban/rural status, geographic region, median education level, poverty prevalence, and race/ethnicity. Referent group was those municipalities who reported "No" or “Don’t know” to having supports for healthy food access within the specified category or had missing values. ^a^ Among 1787 municipalities with a “Yes” or “No” response to the question on food policy councils (excludes “Don’t know”). ^b^ Does not include municipalities who responded “Don’t know” to the question on food policy councils. Boldface prevalence estimates indicate statistical significance (*p*-value < 0.05) based on a Chi-square test for differences between municipalities that reported they have an FPC and those that reported "No". Boldface aORs indicate statistical significance based on the 95% CI not including the null value of 1, comparing municipalities that reported having an FPC to those that reported “No”. The bold indicate statistical significance.

## Data Availability

The data presented in this study are available upon request from the corresponding author. The data are not publicly available due to restrictions.
